# Compartmentalized Polymeric Nanoparticles Deliver Vancomycin in a pH-Responsive Manner

**DOI:** 10.3390/pharmaceutics13121992

**Published:** 2021-11-24

**Authors:** Merve Seray Ural, Mario Menéndez-Miranda, Giuseppina Salzano, Jérémie Mathurin, Ece Neslihan Aybeke, Ariane Deniset-Besseau, Alexandre Dazzi, Marianna Porcino, Charlotte Martineau-Corcos, Ruxandra Gref

**Affiliations:** 1 Institut de Sciences Moléculaires d’Orsay, CNRS UMR 8214, Université Paris-Sud, Université Paris-Saclay, 91405 Orsay, France; merve-seray.ural@universite-paris-saclay.fr (M.S.U.); mario.menendez-miranda@universite-paris-saclay.fr (M.M.-M.); giuseppina.salzano@universite-paris-saclay.fr (G.S.); 2 Institut de Chimie Physique, CNRS UMR 8000, Université Paris Sud, Université Paris-Saclay, 91405 Orsay, France; jeremie.mathurin@universite-paris-saclay.fr (J.M.); ece.aybeke@universite-paris-saclay.fr (E.N.A.); ariane.deniset@universite-paris-saclay.fr (A.D.-B.); alexandre.dazzi@universite-paris-saclay.fr (A.D.); 3 Conditions Extrêmes et Matériaux: Haute Température et Irradiation (CEMHTI), CNRS UPR 3079, Université d’Orléans, 45071 Orléans, France; marianna.porcino@cnrs-orleans.fr (M.P.); charlotte.martineau@uvsq.fr (C.M.-C.); 4 ILV UMR CNRS 8180, Université de Versailles St-Quentin en Yvelines, Université Paris Saclay, 78035 Versailles, France

**Keywords:** nanoparticle, biodegradable polymer, vancomycin, controlled release, drug location, antibiotic

## Abstract

Vancomycin (VCM) is a last resort antibiotic in the treatment of severe Gram-positive infections. However, its administration is limited by several drawbacks such as: strong pH-dependent charge, tendency to aggregate, low bioavailability, and poor cellular uptake. These drawbacks were circumvented by engineering pH-responsive nanoparticles (NPs) capable to incorporate high VCM payload and deliver it specifically at slightly acidic pH corresponding to infection sites. Taking advantage of peculiar physicochemical properties of VCM, here we show how to incorporate VCM efficiently in biodegradable NPs made of poly(lactic-co-glycolic acid) and polylactic acid (co)polymers. The NPs were prepared by a simple and reproducible method, establishing strong electrostatic interactions between VCM and the (co)polymers’ end groups. VCM payloads reached up to 25 wt%. The drug loading mechanism was investigated by solid state nuclear magnetic resonance spectroscopy. The engineered NPs were characterized by a set of advanced physicochemical methods, which allowed examining their morphology, internal structures, and chemical composition on an individual NP basis. The compartmentalized structure of NPs was evidenced by cryogenic transmission electronic microscopy, whereas the chemical composition of the NPs’ top layers and core was obtained by electron microscopies associated with energy-dispersive X-ray spectroscopy. Noteworthy, atomic force microscopy coupled to infrared spectroscopy allowed mapping the drug location and gave semiquantitative information about the loadings of individual NPs. In addition, the NPs were stable upon storage and did not release the incorporated drug at neutral pH. Interestingly, a slight acidification of the medium induced a rapid VCM release. The compartmentalized NPs could find potential applications for controlled VCM release at an infected site with local acidic pH.

## 1. Introduction

The glycopeptide antibiotic vancomycin (VCM) represents the last-line defense against infections caused by numerous species of Gram-positive bacteria including *Staphylococci*, *Enterococci*, *Streptococci*. Those strains are responsible of the majority of bacterial infections in humans worldwide and represent the main cause of hospital-acquired infections [1,2,3]. VCM is thus a gold standard treatment against *Staphylococcus aureus (S. aureus)* and in particular, of the methicillin-resistant *S. aureus* (MRSA) that can invade and survive within different types of cells such as keratinocytes, fibroblasts, enterocytes, and osteoblasts [4,5,6,7] or even alveolar macrophages thus evading the immune system [8,9,10].

VCM exerts its biological action by forming key hydrogen bonds with carboxy-terminal peptide moieties of bacterial cell walls [11]. Despite its efficacy, VCM suffers from several drawbacks mainly related to its poor bioavailability [12], low tissue penetration, and incapacity to bypass cell membranes. Therefore, VCM is mainly administered by the intravenous route as a slow infusion over at least 60 min with a frequency of administration of every 8 to 24 h [13]. However, the low pH (<4) of the VCM infusion leads to tissue irritation [14]. Moreover, VCM is a large, highly hydrophilic and polar compound, which partially ionizes at physiological pH and does not readily diffuse from blood to tissues [15]. It also strongly binds to plasma proteins [16] and is eliminated via renal route [17]. Thus, VCM has to be administered in large doses to reach sufficient therapeutic activity, which in turn leads not only to the emergence of VCM-resistant strains, but also unwanted side effects such as thrombophlebitis, fever, kidney damage, epidermal necrolysis, and a hypersensitivity reaction known as red man syndrome [18,19,20].

In this context, nanotechnology opens new opportunities to improve VCM bioavailability and diminish its adverse effects. Indeed, nanoparticles (NPs) are engineered to protect drugs from degradation and rapid elimination, to allow controlled release and targeted drug delivery to the infection sites [21]. 

Infected tissues are characterized by an acidic pH that can reach to 5.5 due to the accumulation of lactic and acetic acids as a result of low oxygen-triggered anaerobic fermentation [22,23]. Therefore, targeted VCM delivery using pH-responsive NPs could be a pertinent strategy upon fighting with Gram-positive infections while avoiding the drawbacks of conventional VCM treatment [24]. NPs can also be employed as Trojan horses to track *S. aureus* in its hideouts inside cells [21].

An “ideal” VCM nanocarrier should: (i) Be prepared by a simple method, without excipients and using materials with known safety profiles; (ii) efficiently incorporate high drug loading (DL); (iii) have good stability upon storage; and (iv) allow a pH-responsive VCM release specifically at acidic pH, but not in physiological conditions. However, the efficient encapsulation of large hydrophilic molecules such as VCM in NPs is challenging, mainly because the drug’s low affinity for the hydrophobic materials generally used to elaborate NPs [25]. Several attempts have been made to incorporate VCM in NPs. Liposomal formulations showed enhanced MRSA eradication, but their encapsulation efficiency was less than 20% wt% [8,26,27]. Gold and silver NPs combined with VCM showed enhanced antibacterial activities [28,29,30,31,32] but metal NPs were reported to have toxic side-effects and tendency to aggregation [33].

Among the polymer-based biomaterials, food and drug administration (FDA)-approved polylactic acid (PLA) and poly(lactic-co-glycolic acid) (PLGA) have shown clear potency as drug carriers due to their clinically well-established biocompatibility and low toxicity. PLA and PLGA degrade by hydrolysis, forming lactic and glycolic acid metabolites which are eliminated from the body through natural pathways [34]. In previous studies, VCM was incorporated in nano-and microparticles made of PLA and PLGA, followed by a progressive release at physiological pH [35,36,37,38]. For instance, diblock PLGA-poly(ethylene glycol) (PEG) NPs were loaded with VCM up to 8.9 wt% [37]. PLGA NPs designed for oral VCM administration reached payloads of 0.5–7.4 wt% and released the whole VCM cargo within 12 h at physiological pH [39].

Other research groups attempted to achieve a pH-controlled VCM release by using composite NPs prepared by combining several ingredients (PLGA, chitosan, PEG-PLGA and Eudragit E100) [19], by using chitosan cores and PLGA shells [40] or by synthesizing PLGA-b-poly(L-histidine)-b-poly(ethylene glycol) derivatives [23]. Switchable surface charge properties were achieved on VCM-loaded chitosan/polymer core-shell composite NPs [40]. However, DLs only reached around 8 wt%, the preparation methods were sophisticated and specific release of VCM in acidic and not in neutral conditions was difficult to be achieved.

In this context, our strategy was to take advantage of the VCM’s peculiar physicochemical properties (highly pH-dependent global charge, hydrophilicity, tendency to aggregation) in order to engineer NPs with high drug payloads, no release at neutral pH and rapid release in acidic condition. To do so, we used a series of charged PLA and PLGA (co)polymers able to establish electrostatic interactions with VCM in a pH-dependent manner. As a result, we created compartmentalized NPs, with a loading of around 25 wt%, that spontaneously released the whole VCM cargo upon changes from neutral to acidic conditions. 

To decipher the underlying molecular mechanisms, we investigated the effect of polymers’ physicochemical properties (molar mass, chemical composition, nature of the end groups) on VCM loading and release. Noteworthy, the pH-dependent VCM release was achieved by avoiding the use of excipients such as pH-responsive materials.

To achieve the best quality control, the VCM-loaded NPs were characterized at the single NP level to determine their morphology, composition, and respective location of their components. In this context, transmission electron microscopy (TEM) and cryogenic TEM (cryo-TEM) enabled to investigate the NPs’ peculiar internal structures. Electron microscopies (EMs) associated with energy-dispersive X-ray (EDX) spectroscopy and atomic force microscopy coupled to infrared spectroscopy (AFM-IR) allowed to locate VCM in the NP’s compartments. Finally, solid state nuclear magnetic resonance (NMR) spectroscopy brought complementary insights on VCM location in the NPs and pH-dependent interactions between VCM and the NPs. 

## 2. Materials and Methods

### 2.1. Materials

(Co)polymers with different lactic acid: glycolic acid ratios (from 50:50 to 100:0), end groups (carboxylic (C) or ester (E)) and molecular weights (M_w)_ were kindly donated by Sequens (Expansorb, Aramon, France) with the compositions: 

PLGA, 50:50, C terminated (Mw: 42–65 KDa, EXPANSORB^®^ 10P001) named PLGA54C,

PLGA, 75:25, C terminated (Mw: 37–84 KDa, EXPANSORB^®^ 10P002) named PLGA61C, 

PLGA, 75:25, C terminated (Mw: 76–130 KDa, EXPANSORB^®^ 10P003) named PLGA103C,

PLGA, 50:50, C terminated (Mw: 95–130 KDa, EXPANSORB^®^ 10P012) named PLGA113C, 

PLGA, 75:25, C terminated (Mw: 5–20 KDa, EXPANSORB^®^ 10P015) named PLGA13C,

PLGA, 50:50, ester terminated (Mw: 72-91 KDa, EXPANSORB^®^ 10P016) named PLGA82E, 

PLGA, 50:50, C terminated (Mw: 15-40 KDa, EXPANSORB^®^ 10P017) named PLGA28C,

PLGA, 50:50, C terminated (Mw: 10–20 KDa, EXPANSORB^®^ 10P019) named PLGA15C, 

PLGA, 50:50, ester terminated (Mw: 10–20 KDa, EXPANSORB^®^ 10P024) named PLGA15E, 

PLGA, 45:55, C terminated (Mw: 15–30 KDa, EXPANSORB^®^ 10P032) named PLGA23C,

PLA, C terminated (Mw: 10–20 KDa, EXPANSORB^®^ 10P005) named PLA15C, 

PLA, C terminated (Mw: 6–10 KDa, EXPANSORB^®^ 10P006) named PLA8C.

VCM (cancomycin hydrochloride for injection, USP, Sandoz^®^) was kindly provided by the International Center for Research on Infectious diseases (Lyon, France). Poly (vinyl alcohol) (PVA) (30,000–70,000 g/mol, 88% hydrolyzed), sodium cholate, anhydrous dichloromethane (DCM), trifluoracetic acid (TFA), dimethyl sulfoxide (DMSO), hydrochloric acid (HCl), sodium chloride (NaCl), potassium chloride (KCl), sodium phosphate dibasic (Na_2_HPO_4_), potassium phosphate monobasic (KH_2_PO_4_) were all obtained from Sigma-Aldrich. Buffers covering a range of pH from 5.3 to 7.4 were prepared by mixing stock solutions of KH_2_PO_4_ and Na_2_HPO_4_ in distilled water. Complete cell culture medium prepared with DMEM (Life Technologies, Saint-Aubin, France) supplemented with 10% *v*/*v* decomplemented fetal bovine serum (FBS) (Life Technologies, France) and 1% penicillin/streptomycin (Life Technologies, France) was used for release studies. Solvents and reagents were of analytical grade and were used without further purification except otherwise stated. 

### 2.2. Preparation of VCM-Loaded PLA and PLGA NPs 

**Figure 1:**
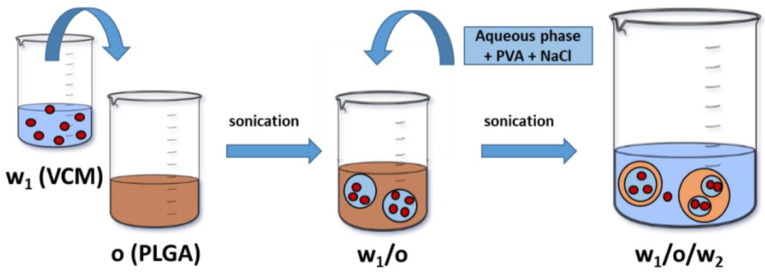
Schematic representation of the preparation of VCM-loaded PLA and PLGA NPs using a double-emulsion (w_1_/o/w_2_) solvent evaporation method. The organic (o) and aqueous (w_1_ and w_2_) phases are represented in brown and blue, respectively. VCM (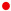) is solubilized in w_1_.

VCM-loaded PLA and PLGA NPs were prepared by a water-in-oil-in-water (w_1_/o/w_2_) emulsion solvent evaporation method using PVA as an emulsifier as represented schematically in Figure 1. Briefly, 0.2 mL of an aqueous phase containing VCM (150 mg/mL) in 1 mL of DCM containing PLA or PLGA (75 mg) was sonicated using a probe (Sonopuls HD 2070, Bandelin electronic GmbH & Co, Berlin, Germany) at 40% of power for 15 s. To create a w_1_/o/w_2_ emulsion, 5 mL of 0.5% *w*/*v* PVA (or sodium cholate) solution containing or not 1% *w*/*v* NaCl was poured to the first emulsion, followed by an immediate sonication for 30 s at 40% of power in an ice bath to avoid overheating. The resulting w_1_/o/w_2_ emulsion was left at room temperature under gentle magnetic stirring until complete evaporation of the organic solvent. Control plain NPs were prepared in the same conditions except that VCM was not added. All formulations were prepared at least in triplicate.

### 2.3. Drug Quantification

The drug loading (DL) calculated as shown in Equation (1) is defined as the mass fraction of a NP that is composed of drug. The entrapment efficiency (EE) (Equation (2)) is defined as the fraction of drug effectively encapsulated into the NPs compared with the amount of drug that was used to prepare the NPs.

(1) $$\mathrm{DL}\;(\%)=\frac{\left(mg\;of\;encapsulated\;drug\right)}{\left(mg\;of\;polymer\right)}\times 100$$

(2) $$\mathrm{EE}\;(\%)=\frac{\left(mg\;of\;encapsulated\;drug\right)}{\left(mg\;of\;drug\;initially\;added\;to\;the\;formulation\right)}\times 100\;$$

The DL was determined by two methods: (i) an indirect method, analyzing the non-incorporated drug in the supernatants after NP centrifugation and (ii) a direct method, dissolving the NPs (pellet) and quantifying the extracted drug. The DLs and EEs calculated through both methods were consistent with less than 5% differences, validating the method. 

VCM was quantified by reverse-phase liquid chromatography, using a C18 column (Kinetex 5u C18, 100A, Phenomenex, Le Pecq, France) connected to a HPLC (Agilent 1100, Agilent Technologies, Les Ulis, France) system with a UV-vis detector (wavelength was fixed at λ = 254 nm). The chromatographic conditions are detailed in the Table S1.

### 2.4. Determination of Residual PVA in the Preparation of PLGA and PLA Particles 

The residual PVA in the NPs was quantified by a colorimetric assay based on the formation of a blue complex between PVA and iodine in the presence of boric acid, as detailed in SI [41]. All experiments were prepared in triplicate.

### 2.5. Release Studies

VCM release studies were performed in complete cell culture medium (DMEM supplemented with 10% fetal calf serum (FCS), 1% penicillin/streptomycin and 1% L-glutamine) at pH 7.4 and 4.5, and also in PB 100 mM at pH 5.3, 6.4, and 7.4. In all cases, the NP suspensions were diluted ten times with the release media. After incubation at 37 °C under stirring, the NP suspensions were centrifuged at 17,000× *g* for 15 min and the supernatants were recovered. VCM release was assessed by HPLC-UV-VIS as described in Table S1 and expressed as % referring to the DL of the formulations. All experiments were carried on at least in triplicate. 

### 2.6. Nanoparticle Characterization

#### 2.6.1. NP Size Measurement by Dynamic Light Scattering (DLS) and Zeta Potential Measurements

The NPs’ average hydrodynamic diameter was determined by DLS at 25 °C with an equilibration time of 60 s using a Zetasizer^®^ (Nano ZS90, Malvern Instruments, Worcestershire, UK). All formulations were measured at least in triplicate. Mean diameters were reported as Z average (nm) ± SE (standard error) with the polydispersity index (PDI). The NPs mean diameters were monitored up to three months of storage at 25 °C. The NPs Zeta potential was measured in KCl 1 mM using a Zetasizer.

#### 2.6.2. Solid State NMR Spectroscopy

The ^1^H magic-angle spinning (MAS) NMR spectra were recorded at a magnetic field of 20 T, using a Bruker 850 MHz WB NMR spectrometer and using a HXY 1.3 mm probe in double mode. The spectra were acquired using Hahn echo pulse sequence, with a 90° pulse duration of 1.4 μs, an inter-pulse delay synchronized with one rotor period, and a spinning rate of 60 kHz. The recycle delay was set to 20 s and 16 transients were recorded for each sample. The ^1^H chemical shifts were referenced to tetramethylsilane (TMS). The assignment of VCM protons was made by comparison with liquid NMR spectra. 

The ^13^C cross-polarization (CP) experiments were recorded on a 9.4 T magnet (^1^H and ^13^C Larmor frequency of 400 and 100 MHz, respectively) with a Bruker spectrometer, using a 4 mm double resonance MAS probe. The ^13^C chemical shifts are referenced to adamantane. The contact time was set to 3.5 ms, the recycle delay was set at 5 s, the initial 90° pulse on ^1^H to 3 μs with a radio frequency (RF) field of 83 kHz. ^1^H SPINAL-64 decoupling was applied during the ^13^C acquisition. The NMR spectra were acquired using TopSpin 3.5 Bruker Software and processed with DM fit program [42].

#### 2.6.3. Size-Exclusion Chromatography (SEC)

The polymer molar masses were determined after release using SEC with THF as eluent (1 mL/min) at room temperature. SEC was equipped with a refractive index detector (Waters 410) and two mixed-bed ViscoGELTM columns (7.8 × 300 mm, type GMHH R-H) provided by Viscotek. 

#### 2.6.4. TEM and Cryo-TEM Investigations

TEM images were acquired on a JEOL electron microscope (JEM 100 CX II, operating at 120 kV) equipped with an US1000 2k × 2k camera. To do so, 5 µL of samples were deposited onto a 400 mesh carbon-coated copper grid beforehand treated by glow discharge, and dried with a filter paper after 30 s Cryo-TEM images were acquired on a JEOL 2200FS (Jeol, Croissy, France) energy-filtered (20 eV) field emission gun electron microscope operating at 200 kV. To do so, 5 μL of NPs suspensions were deposited onto a 200 mesh copper grid and flash-frozen in liquid ethane cooled down at liquid nitrogen temperature. 

#### 2.6.5. Scanning Electron Microscopy (SEM)-EDX

SEM-EDX quantitative analysis was performed using a Carl Zeiss (Croissy, France) SEM with a field emission gun equipped with secondary electron detectors. SEM was coupled with an EDX equipped with a 50 mm^2^ SDD X-Max detector from Oxford Instruments (Gometz-la-Ville, France). The characterization of the NPs was performed at 2 keV.

#### 2.6.6. Scanning TEM-EDX

Chemical composition analyses were performed with a scanning TEM (STEM) device equipped with a high angle annular dark field (HAADF) imaging. The microscope was composed of a field emission gun, a scanning device, an EDX detector and an electron energy loss spectrometer. Carbon films on 400 Mesh Copper Grids were treated by glow discharge cleaning system to make the film hydrophilic. About 5 μL of samples were deposited onto a carbon-coated copper grid for 1 min, and then the excess of liquid was removed. The characterization of the NPs was performed at 200 keV.

#### 2.6.7. Fourier Transform IR (FTIR) Spectroscopy

Attenuated total reflection (ATR) FTIR spectra of NPs components were obtained by using a Bruker Vertex 70 FTIR spectrometer, Bruker, Palaiseau, France (MCT detector, cooled down using liquid nitrogen) and a ATR module (PIKE MIRacle crystal plate diamond ZnSe). Spectra were recorded in the range of 4000–600 cm^−1^ with an accumulation of 100 scans and a resolution of 4 cm^−1^. Only significant absorptions were listed.

#### 2.6.8. AFM-IR

Aliquots of the NPs samples were deposited on CaF_2_ cell windows and left to dry completely over several days. Experiments were performed with either NanoIR-2 or NanoIR-2s (Anasys Instruments, Goleta, CA, USA) coupled with a multichip QCL source (Daylight Solutions; tunable repetition rate range of 0–2 MHz; spectral resolution of 0.1 cm^−1^) covering the range from 1900 cm^−1^ to 900 cm^−1^ of the mid-IR region with a tunable repetition rate. An Au-coated silicon tapping AFM-IR cantilever, Watsonville, CA, USA (Multi75GB-G, f = 75 kHz 3 N/m, Budget Sensors) was employed. Gold coating was used to avoid effects linked to IR absorption of the silicon cantilever. Power was set between 22% and 100% with tapping mode frequency around 50–60 kHz. Laser pulse rate was set around 317 kHz with a pulse duration of 200 ns. Topography and IR maps were processed using the Mountains Map 7.3 software and the spectra (sampled at 1 cm^−1^) were filtered using the Savitzky–Golay algorithm (1rd order polynomial using 11 points) [43]. 

## 3. Results and Discussion

### 3.1. NP Preparation

As previously shown by a set of studies involving NMR spectroscopy, X-ray crystallography and molecular modeling, VCM exerts its biological action by: (i) specific complexation with peptides containing terminal D-alanyl-D-alanine units on the surface of bacterial cell walls leading to stable hydrogen bond interactions [44] and (ii) establishing charge–charge interactions with peptide carboxylate groups [45,46,47]. This is in line with a previous study showing that VCM was associated to nanodiamonds only when electrostatic interactions were established, and more particularly, on the surfaces comporting carboxyl groups [48]. Those observations inspired us to use a series of biodegradable PLGA and PLA (co)polymers possessing carboxyl end groups, to establish electrostatic interactions with VCM. This will enhance the payloads and help achieving a pH-triggered release. Additionally, (co)polymers, with ester end groups, were used as controls. The (co)polymers are named here PL(G)A_x_C or PL(G)A_x_E; C and E represent respectively the carboxyl and the ester end groups whereas x represents the mean M_w_.

Several methods are employed to load hydrophilic drugs, comprising processing through supercritical fluids and emulsification procedures [49]. In this study, a double-emulsion solvent evaporation method schematically represented in Figure 1 was employed to prepare the NPs and to maximize the interactions at the interfaces between aqueous and organic phases. The double-emulsion method was used since 1998 to incorporate large hydrophilic macromolecules such as proteins inside NPs [50] and more recently for VCM incorporation [39,51]. Briefly, in our study, an aqueous VCM solution was dispersed in an organic phase containing the PLA or PLGA (co)polymers resulting in the first emulsion. Sonication was employed to efficiently reduce the aqueous droplet size [52,53]. The hydrophilic carboxyl chain ends of the (co)polymers tended to migrate toward the inner aqueous phase where VCM was located. Then, a water-in-oil-in-water (w_1_/o/w_2_) emulsion was prepared forming of oil droplets containing aqueous compartments with VCM, followed by the evaporation of the organic solvent that led to polymer precipitation and NPs formation. (Figure 1).

**Figure 2:**
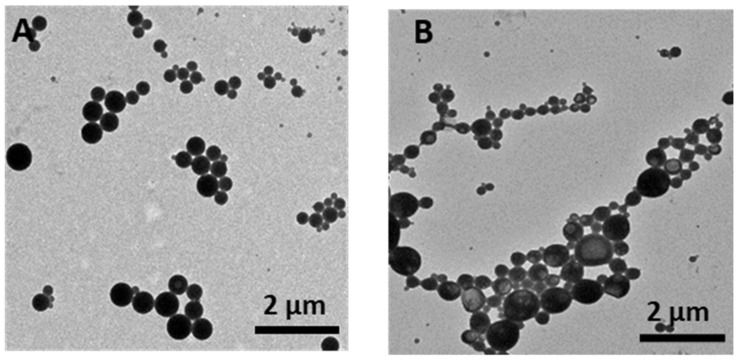
Typical TEM images of NPs prepared by double-emulsion using PLGA_15_C: (**A**) empty and (**B**) VCM-loaded.

Different families of PLA and PLGA (co)polymers were evaluated for VCM incorporation in NPs. The characteristics of each polymer and the corresponding NP formulation are shown in Table 1. Polymers differ in end group, composition, and M_w_ that determine the inherent viscosity and the performance to encapsulate hydrophilic drugs. As shown in Table 1, all the polymers formed NPs with PDIs lower than 0.26. The mean hydrodynamic diameters determined by DLS ranged from 300 to 370 nm (a typical example of size distribution is given in Figure S1). The increase in NP size was associated with the increment in the M_w_ of the employed polymer: PLGA_15_C (325 ± 25 nm), PLGA_61_C (350 ± 25 nm), and PLGA_113_C (367 ± 25 nm) (Table 1), while drug loading had no significant impact on the NP size. The morphology of the NPs was investigated by SEM (Figure S1) and TEM (Figure 2) and the obtained average sizes corroborated with DLS results (Table 1). Typical TEM images showed that the NPs were spherical and devoid of aggregates. Sizes ranged in between around 100 and 400 nm. 

The NPs’ Zeta potential was close to zero (Table 1), indicating a neutral surface charge which could be attributed to the presence of the non-charged PVA emulsifier. Indeed, PVA can intermingle with the PLA and PLGA chains and stay associated with the NPs’ surface despite an extensive washing procedure [41]. Here, the residual PVA amount was 6 ± 1 wt%, whatever the polymer used, confirming that PVA forms a protective steric layer at the NP surfaces shielding the negative charges of the PLGA and PLA polymer end groups.

### 3.2. Drug Loading and Mechanism

Although all the NP formulations presented similar physical properties such as size, PDI, and Zeta potential, they differed significantly in terms of DL and EE (Table 1). DL ranged from <1 wt% (undetectable) to 14 wt% and EE from <1 wt% to 36 wt%. As a general trend, (co)polymers with the highest M_w_ such as PLGA_54_C and PLGA_113_C did not incorporate VCM, whereas PLGA_15_C and PLGA_23_C (co)polymers with the same compositions but lower M_w_ entrapped a higher amount of drug (14 ± 4 wt% and 9 ± 1 wt% VCM, respectively). Furthermore, ester-terminated polymers failed to incorporate VCM whatever their M_w_. Finally, the higher the hydrophilicity of the (co)polymer (i.e., the lower the L content), the higher the VCM DL. For example, at similar M_w_, PLGA_15_C with 50% L units has a DL of 14 wt%, whereas PLGA_13_C with 75% and PLA_15_C with 100% L units, have DLs of 5 wt% and <1 wt% respectively.

In conclusion, the DL and EE were related to three properties of PLA and PLGA (co)polymers: (i) the M_w;_ (ii) the terminal groups (C or E) and (iii) the percentage of L units. The optimal (co)polymer for VCM incorporation was PLGA_15_C, with the lowest M_w_ and a carboxy end group. From the PLA series, PLA_8_C was the best with 7 ± 2 wt% DL and 16 ± 5 wt% EE. The NP preparation parameters and optimizations are detailed in Tables S2 and S3 with the emphasis of using NaCl to form rapidly the NPs.

**Figure 3:**
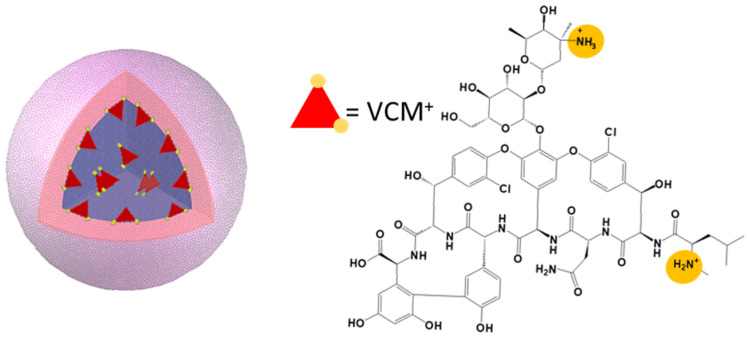
Schematic representation of the hypothesized mechanism of VCM (red triangle) incorporation in a NP compartment (blue) and chemical structure of VCM (protonated form). The VCM-protonated amine groups (in orange) interact with deprotonated carboxylic acid endgroups of the PLGA polymer which are pointing to the interface of aqueous inner compartments.

In a nutshell, the results in Table 1 supported our hypothesis that VCM is efficiently incorporated if it establishes electrostatic interactions with the carboxyl end group of the polymer. Keeping in mind that the same amount of polymer was used to prepare NPs, the lower the M_w_ (i.e., the shorter the polymer chains), the higher the number of carboxy end groups, so the higher the possibilities of electrostatic interactions with VCM. More precisely, during the formation of the first emulsion (Figure 1), electrostatic interactions occur depending on the physicochemical properties of the VCM and the (co)polymers used to incorporate it. The VCM solutions have an acidic pH (~4) at which it has an overall positive charge arising from its two positively charged alkylamine groups and a negatively charged carboxylic acid group [54,55], while PLA and PLGA, with their carboxyl end groups (pKa = 3.85), are slightly deprotonated [56]. Therefore, charge–charge interactions can be established between the (co)polymers and the VCM at the interface as schematized in Figure 3. Drug loading can be ensured as the VCM will have less tendency to leak out of the droplets. In addition, VCM self-association in the aqueous compartments should also contribute to the formation of bulky structures, favoring their loading.

**Figure 4:**
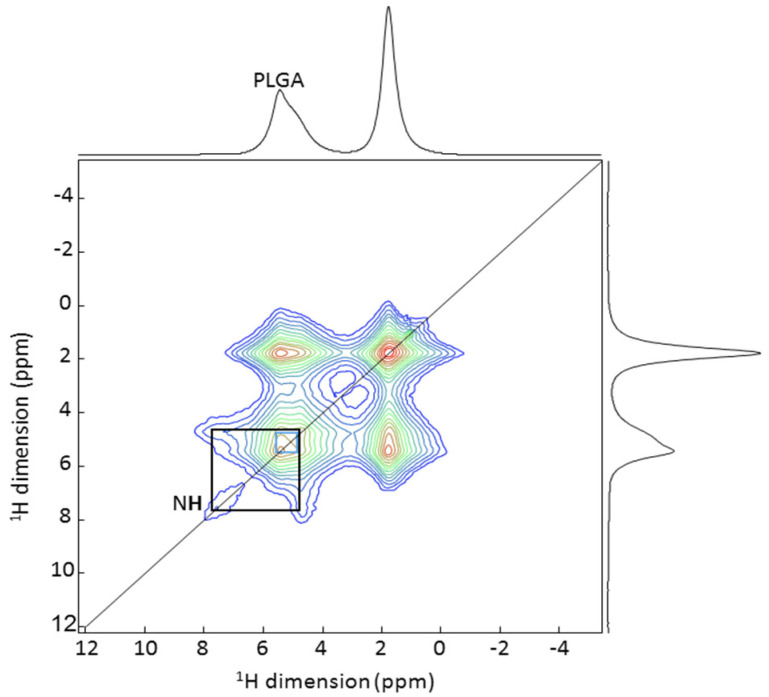
2D ^1^H-^1^H MAS NMR spectrum of PLGA_15_C NPs loaded with VCM. The black rectangle highlights the cross-correlation peaks between the NH of the VCM and the protons of the PLGA.

Solid state NMR spectroscopy was used to provide further information about the mechanism of the VCM encapsulation since it allows a deep characterization of the structure and interactions between the drug and the polymer carrier at an atomic level [57]. At first, ^1^H and ^13^C MAS NMR spectra of empty and VCM-loaded PLGA_15_C NPs were recorded (Figure S2). The presence of the ^13^C resonances of VCM in the loaded particles confirmed the successful VCM encapsulation. Next, the ^1^H-^1^H 2D MAS NMR was performed to investigate the interactions between drug and the polymer. Figure 4 presents the correlation study including cross peaks between VCM and PLGA. Despite the use of high magnetic field (20 T) and fast-MAS (60 kHz), there was still a strong overlap of the proton NMR signals of the NPs and VCM. Only a NH proton (8 ppm) and an OH proton (9 ppm) of VCM could clearly be resolved. Cross-correlation peaks obtained between one NH from the VCM and the protons from PLGA indicate close spatial proximity between the drug and the polymer, hence the electrostatic interactions between these two. This assessment is in agreement with NMR studies in the literature that show the strong interactions of NH amide groups of the VCM with the terminal carboxylate of the bacterial cell wall peptide [58].

**Figure 5:**
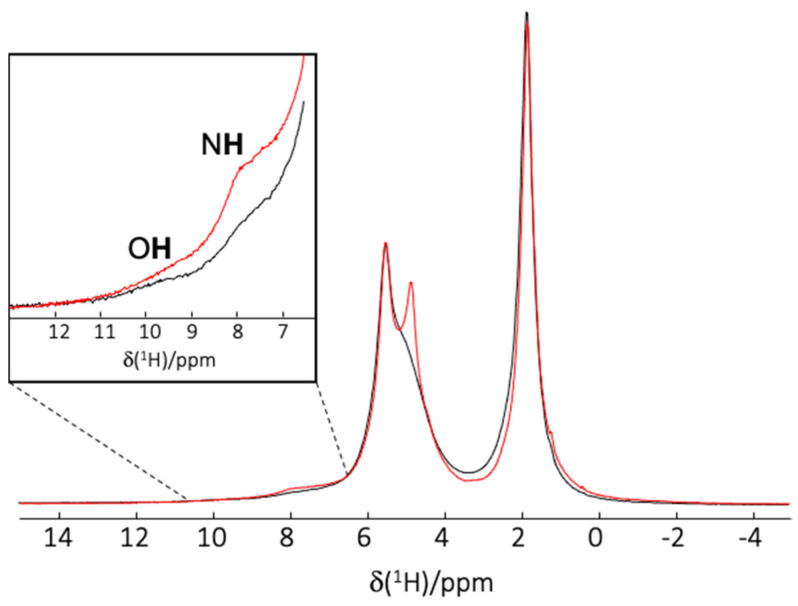
^1^H MAS NMR spectra of VCM-loaded PLGA_15_C NPs prepared in H_2_O (black spectrum) and in D_2_O by using deuterated VCM (red spectrum). An expansion on OH and NH of VCM is shown in insert.

To give further proof of the electrostatic interactions, additional experiments were carried out. VCM-loaded NPs were prepared by using deuterated water and deuterated VCM in order to shield the electrostatic interactions between VCM-polymer. As awaited, results showed dramatical modifications due to the lack of H bond interactions: DL and EE decreased from 14 ± 4 to 5 ± 1 wt % and from 36 ± 2 to 14 ± 1 wt%, in the case of non-deuterated and deuterated systems, respectively. Eventually, in order to detect the ^1^H species by NMR, the deuterated VCM-loaded NPs were submitted to a back exchange from D to H by prolonged contact with water. Results are summarized in Figure 5. The deuterated formulation (red spectrum) in the ^1^H MAS NMR spectrum presents narrower NH proton resonances of VCM (i.e., they appear more intense, despite the lower DL), indicating weak interactions between the polymer and VCM. As control, VCM-loaded NPs prepared in water showed broader NH proton signal (black line in Figure 5), which clearly evidences the existence of H bond interactions between VCM and the polymer. These H-bonds indeed result in ^1^H chemical shift distribution (distribution of H-bonds) hence broader line in the spectrum. In conclusion, the establishment of VCM-polymer H bonds were shown to be responsible for high DL and EE.

Knowing that the electrostatic interactions between VCM (monomers or self-associates) and polymer play a major role, and are governed by the pH, a study has been carried out on observing the effect of the pH of the inner and outer aqueous phases used in the 1st and 2nd emulsion respectively during preparation of VCM-loaded NPs (Table 2). Neither the DL nor the EE depended on the pH of the outer medium in a range from 6.3 to 8.5. On the contrary, when the pH of the inner VCM solution (1st emulsion) was 7.4, both the DL and the EE were increased reaching 25 ± 3 wt% and 53 ± 7 wt% respectively. The viscosity of the VCM solution was increased at pH 7.4 as compared to pH 4.0. Presumably, VCM self-association/aggregation occurred preventing VCM from leaking out of the inner droplets during NP preparation thus resulting in improved DL and EE (Table 2).

In conclusion, from the series of PLA and PLGA (co)polymers studied here, PLA_8_C and PLGA_15_C were the best studied materials for the VCM encapsulation. They had the lowest M_w_ and carboxyl end groups. The optimization study reported a dramatic enhancement of VCM loading into PLGA_15_C NPs with DL and EE up to 25 wt% and 53 wt% respectively, which to the best of our knowledge, overcome the DLs and EEs reported so far for VCM incorporation in other NPs or in liposomes [8,26,27,39].

Finally, when the NPs were stored at 4 °C for three months, their mean diameter remained unchanged (less than 5% variation) and less than 4% of the incorporated VCM was released over this time. These results indicate a good stability upon storage. 

### 3.3. NPs’ Inner Morphology

**Figure 6:**
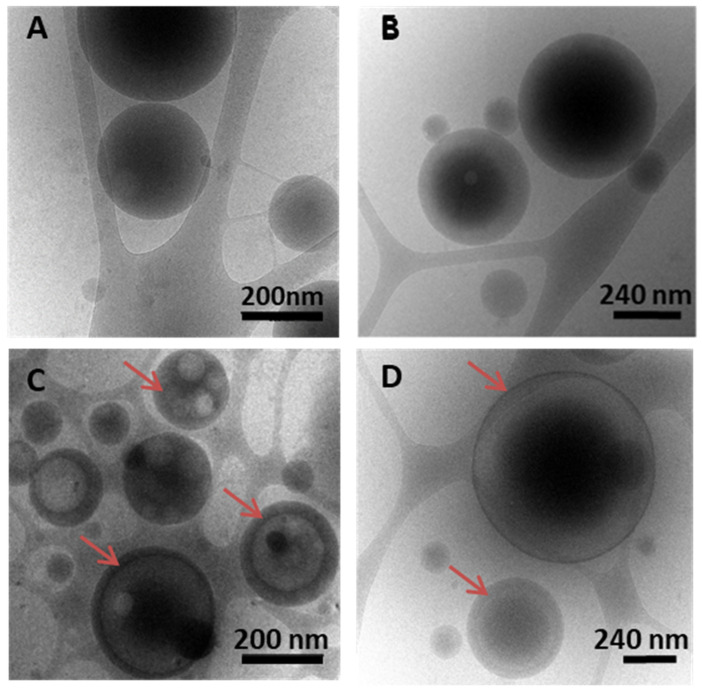
Representative cryo-TEM images of (**A**) empty PLGA_15_C NPs; (**B**) empty PLA_8_C NPs; (**C**) VCM-loaded PLGA_15_C NPs; and (**D**) VCM-loaded PLA_8_C NPs. Polymer shells are indicated with red arrows.

Cryogenic TEM (cryo-TEM) investigations were carried on to gain insights into the inner structure of the PLGA and PLA NPs. Interestingly, the VCM-loaded PLGA_15_A and PLA_8_C NPs possessed relatively large inner aqueous compartments (lighter regions) delimited by thin polymer shells (darker regions) of around 10–40 nm as pointed out with red arrows in Figure 6C,D. To the best of our knowledge, this is the first time that large compartments inside PLA or PLGA-based NPs are visualized. On the contrary, empty NPs (prepared by the same procedure, but without VCM) were dense and devoid of compartments (Figure 6A,B). This striking difference in the inner structure between loaded and empty PLGA and PLA NPs reinforces the fact that VCM-polymer interactions happen in the NP inner aqueous compartments and stabilize these compartments favoring the VCM encapsulation.

### 3.4. Individual NP Characterization: Localization of VCM

#### 3.4.1. SEM-EDX

SEM equipped with EDX is a method of choice to investigate NPs individually, analyzing both their morphology and composition. It commonly identifies the elemental composition on the NPs’ top layers (less than 10 nm depth) since the electron beam has energy of only 2 keV, hampering its penetration inside the cores. PLGA and VCM are composed of carbon, hydrogen, and oxygen atoms whereas VCM possess also nitrogen atoms. Therefore, nitrogen detection by SEM-EDX gives information on VCM presence in the NPs’ top layers. 

**Figure 7:**
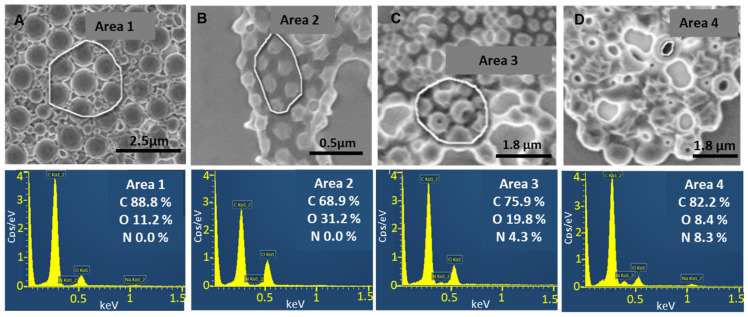
SEM images (top) with EDX analysis (below) of (**A**) empty PLGA_15_C NPs, (**B**) VCM-loaded PLGA_15_C NPs. Sample B was continuously analyzed until holes appeared due to polymer melting (**C,D**) and EDX analysis was further focused on the inner regions which were thus revealed.

Figure 7 presents the EDX analysis of PLGA_15_C NPs, loaded or not with VCM. Elemental analysis was performed on the areas indicated in SEM images. As expected, no trace of nitrogen was detected in empty NPs (Figure 7A). This was the case also with VCM-loaded NPs suggesting that VCM was embedded deeper in their cores (Figure 7B). The SEM observations were pursued until the formation of holes in their structure (Figure 7C,D). Interestingly, EDX elemental analysis focused on selected areas of the holes revealing the presence of 4.3% nitrogen (Figure 7C) and up to 8.3% when only the hole was analyzed (Figure 7D). These investigations of individual NPs rule out the presence of VCM on the NP surface and support VCM loading inside their cores.

#### 3.4.2. STEM-EDX

**Figure 8:**
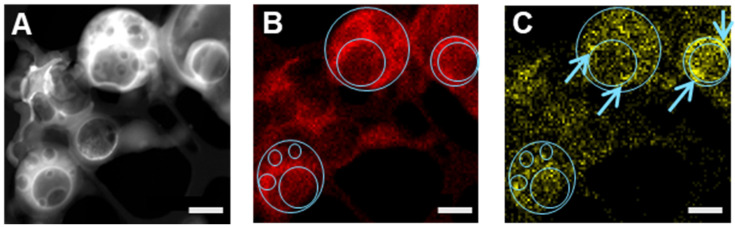
(**A**) STEM image of VCM-loaded PLGA_15_C NPs in which inner aqueous compartments are visible. Chemical mapping for elemental analysis of (**B**) carbon (red) and (**C**) nitrogen (yellow). Blue lines are guides for the eyes and blue arrow indicates nitrogen-rich regions. Scale bars are 500 nm.

Further characterization of individual NPs was performed by STEM-EDX that enables qualitative and semi-quantitative measurements of the atomic compositions, as the beam passes throughout the sample. Therefore, in contrast to SEM-EDX, information is accessible also from the core of the NP. A typical STEM image of VCM-loaded PLGA_15_C NPs (Figure 8A) clearly shows the presence of numerous compartments of NPs similar to the Cryo-TEM ones (Figure 6C).

The corresponding EDX maps of chemical elements highlights the repartition of carbon and nitrogen in VCM-loaded PLGA_15_C NPs (Figure 8B,C). As shown in the elemental mapping of nitrogen in Figure 8C, VCM location can be tracked inside the NPs and mainly at the interface of the water inclusions (blue arrows). This observation supported the suggested VCM encapsulation mechanism based on the establishment of electrostatic interactions at the polymer/drug interfaces.

**Figure 9:**
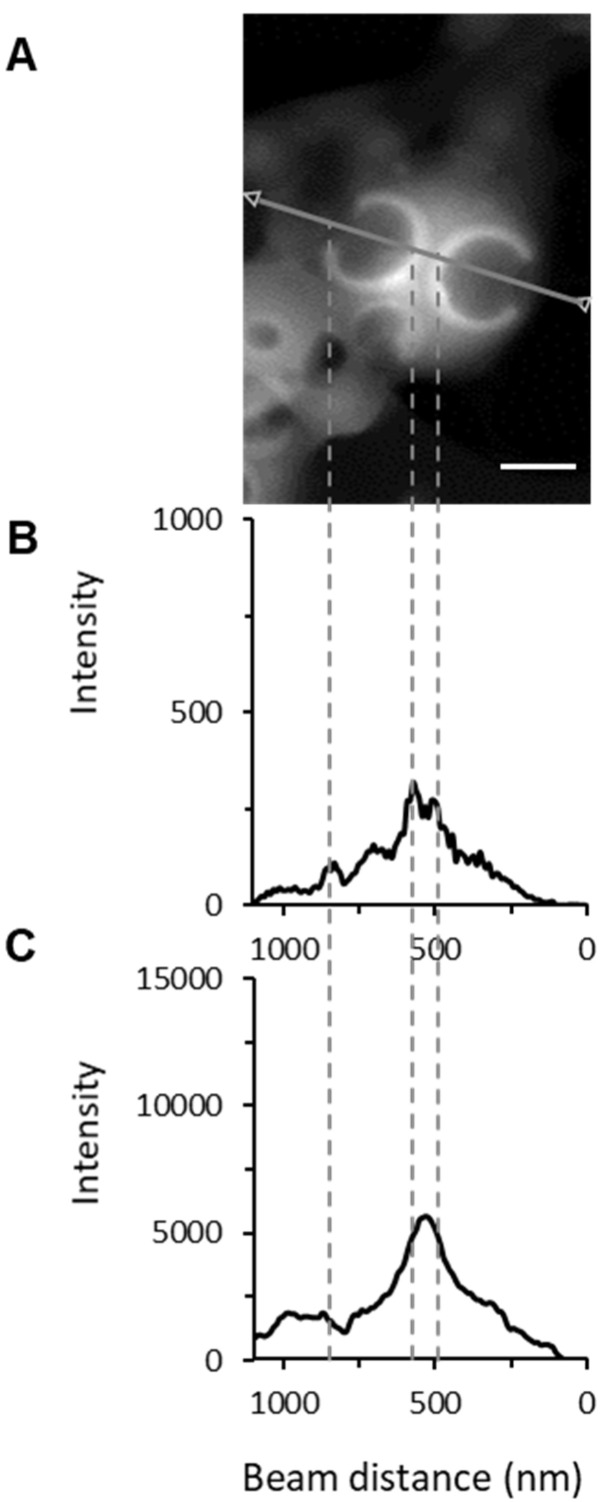
(**A**) STEM image of VCM-loaded PLGA_15_C NPs, EDX line scanning profiles on (**B**) the elemental mapping of the nitrogen and (**C**) of the carbon. The higher values of the nitrogen intensity are indicated by the dashed lines. Scale bar is 250 nm.

Line-scanning profiles of NPs were also recorded to evaluate by EDX the composition distribution on the cross section of the selected line on a single NP with two large compartments (Figure 9). Carbon concentration was significantly lower within the inclusions in line with the presence of less material (Figure 9C). No nitrogen signal corresponding to VCM was detected outside the NPs or onto their surface, in agreement with the SEM-EDX experiments (Figure 7). Noticeably, peaks of nitrogen intensity were observed at the border of the internal compartments (Figure 9B). Similar findings were displayed in Figure S3. In a nutshell the results suggest the preferential accumulation of VCM at the interface and inside of the inclusions.

#### 3.4.3. AFM-IR

**Figure 10:**
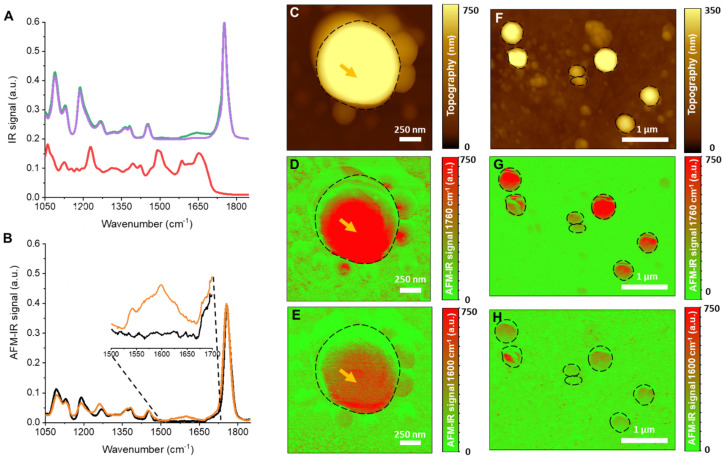
(**A**) ATR-FTIR spectra of VCM (red), empty PLA_8_C NPs (purple), VCM-loaded PLA_8_C NPs (green). (**B**) Local IR spectrum obtained by AFM-IR on empty (black) and VCM-loaded (orange) PLA_8_C NPs at the position indicated with an orange arrow in (**C**–**E**). (**C**,**F**) Topography images of PLA NPs. (**D**,**G**) Tapping AFM-IR map at 1760 cm^−1^ corresponding to the strong absorption of PLA. (**E**,**H**) Tapping AFM-IR map at 1600 cm^−1^ corresponding to VCM absorption.

To complete the compartmentalized NP characterization, we used high resolution vibrational spectroscopy AFM-IR which combines the spatial resolution of AFM (down to around 20 nm) with the capability of IR spectroscopy to unambiguously identify the chemical composition of an individual NP [59,60,61]. Recently, our teams used AFM-IR to locate a hydrophobic drug in PLA NPs [43]. Despite the low loading (<1 wt%), the drug could be well detected and it was shown that it was located only in the NPs’ top layers and not within their cores. This sensitive method was used here to study the VCM location within PLA_8_C NPs. AFM-IR enabled to acquire either an IR chemical map at a specific wavenumber (IR mapping) or an IR spectrum at one specific location in the sample (local IR spectrum). To do so, first, specific wavenumbers were defined as “fingerprints” to unambiguously identify each component of the NP system, the PLA, the VCM, and the PVA. The characteristic IR bands of these three components were recorded by FTIR within the 1850–1000 cm^−1^ range as the accessible IR region in AFM-IR (Figure 10A). The PLA has a main characteristic band at 1760 cm^−1^, corresponding to the stretching of the C=O of carboxyl groups, and other signals at 1068 and 1133 cm^−1^ (CH_3_ groups), 1090 and 1188 cm^−1^ (C-O-C ester bond stretching), and in the region 1300–1500 cm^−1^ (C-H bending of CH_2_ and CH_3_) [62]. The VCM showed a C=O stretching absorption band centered at 1658 cm^−1^, the band associated to the N−H bending at 1502 cm^−1^, an absorption at 1228 cm^−1^ (phenols), and the band associated to the C−N stretching vibrations of amine groups at 1126 and 1060 cm^−1^ [63]. To unambiguously detect the VCM within the NP samples, its absorption at 1600 cm^−1^ was chosen with no interference signal from the other NPs components (PLA, PVA or the residual liquid water) (Figure 10A). Reciprocally, the specific band 1760 cm^−1^ of the PLA_8_C polymers where no VCM and PVA absorption occurred was used to locate the NPs.

As an example, Figure 10C shows a representative AFM topography image recorded on a single NP and the respective IR mapping of PLA (Figure 10D) and VCM (Figure 10E). The signal corresponding to PLA overlapped well with the topography images except at the upper part edges of the NP, which corresponds to the previously reported phase shift effect occurring in the case of PLA NPs [43]. Remarkably, chemical mappings of VCM showed that the drug was well located within the NP and not at its surface (Figure 10E). This result is in contrast with the previous AFM-IR investigations reporting the location of drugs at the PLA NPs’ surface and resulting in uncontrolled release [43].

The final proof of VCM location was given by acquiring local spectra within the NPs’ cores with the excellent AFM resolution. The spectra obtained from the point indicated with orange arrow (Figure 10B) looks very similar to the FTIR one of VCM-loaded PLA_8_C NPs (Figure 10A). 

To complete the study, a region of interest containing several VCM-loaded NPs was investigated (Figure 10F–H). The topography image (Figure 10F) showed round-shaped NPs with sizes between 240 and 500 nm. PLA mapping (Figure 10G) matches with the topography (Figure 10F) showing that even the composition of the smallest NPs (240 nm) could be investigated by AFM-IR. The signal of the largest NPs was more intense, in agreement with the fact that they contained higher amounts of PLA.

In contrast to PLA, VCM mapping showed an uneven drug distribution within the NPs (Figure 10H) and in some cases, a compartmentalized location of VCM. Interestingly, some of the smallest NPs did not contain VCM. It was previously shown that the largest NPs have higher content of inner compartments and some small NPs are dense and not compartmentalized (Figure 6 and Figure 8). Therefore, it can be hypothesized that the smallest NPs do not entrap VCM because there is no internal interface where electrostatic interaction could take place. 

In conclusion, there was a good agreement between AFM-IR, CryoTEM, SEM-EDX, and STEM-EDX investigations which showed that VCM was incorporated inside the NPs’ cores and that it was mainly localized at the interface between the polymer matrix and the inner compartments. 

### 3.5. pH-Controlled Drug Release and Related Mechanism

**Figure 11:**
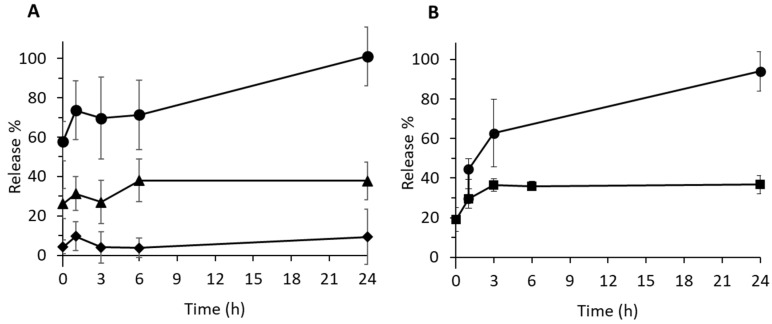
(**A)** pH-dependent VCM release profiles of PLGA_15_C NPs in 100 mM PB at pH = 5.3 (circle), pH = 6.3 (triangle), and pH = 7.4 (diamond). (**B**) VCM release profile of PLGA_15_C NPs in RPMI cell culture medium containing 10% serum (square) at pH 7.4. In another set of experiments, the pH was changed from 7.4 to 5 (circle). All experiments were performed in triplicate at 37 °C.

Drug release from the compartmentalized VCM-loaded NPs was studied as a function of the pH of the release medium (from 5.3 to mimic infection site to 7.4 which correspond to normal physiological conditions) (Figure 11A). At pH 7.4, no more than 10% of the VCM cargo was released within 24 h. In contrast, at pH 5.3, VCM was rapidly released (60%) within the first minutes upon incubation. A total release was observed after 24 h. At pH 6.3, this release shows an intermediate profile. 

Furthermore, the VCM release was studied over 24 h in cell culture media containing serum (Figure 11B). Around 20% of the incorporated drug were released immediately, reaching a plateau at 35% after 3 h. This shows that the majority of the drug (65%) remained associated to the NPs. The same experiment was repeated, but one hour after the start of the release, the pH of the cell culture medium was adjusted to 5. It was found that immediately after, 45% of the entrapped VCM was released, followed by a total release within 24 h. These data show that VCM release can be triggered by acidic conditions in complex release media. 

It is noteworthy that no degradation of the polymers was observed during the timeframe of the release experiments (Figure S4). This suggests that VCM release relies on a diffusion process of the VCM and not on the degradation of the NP matrix. In line with these findings, cryo-TEM observations showed that the compartmentalized structure of the NPs was maintained during drug release whatever the pH of the media (Figure S5). Thus, we hypothesize that VCM release mechanism is based on diffusion through the polymeric matrix and the pH-dependent reversible aggregation of VCM [64]. Indeed, at pH 7.4, VCM self-associates forming bulky structures that diffuse less than the monomeric forms at acidic pH. 

The pH-dependent release behavior of the compartmentalized NP system could be an asset to achieve VCM release specifically in acidic conditions corresponding for example to infected sites or to intracellular compartments in infected cells [21] but not in healthy tissues and organs [65,66].

## 4. Conclusions

This study highlights the importance of the choice of the polymeric material to engineer NPs which can allow efficient VCM incorporation by a simple method. Taking advantage of the strongly pH-dependent physico-chemical properties of VCM (charge, reversible aggregation), it was possible to efficiently incorporate it in biodegradable NPs, by establishing electrostatic interactions, as shown by solid state NMR studies. The drug molecules were confined in large inclusions inside the NPs and their loadings reached 25 wt%. The NPs were comprehensively characterized at the single NP level by using a set of complementary methods including electron microscopies coupled with EDX and AFM-IR. The methodology proposed here for NP physicochemical characterization sets up the basis for a deep understanding not only of the morphology, but also of drug location inside polymer matrices, which in turn has a deep impact on drug release mechanism. Furthermore, the release of the VCM was triggered by acidic environment. These characteristics suggest potential therapeutic applications of targeted VCM delivery at an infected or inflammatory site. The VCM-loaded NPs could be used to fight *S. aureus* infections, *S. aureus* biofilms, or intracellular pathogens. Intravenously administered, formulations could find applications in the treatment of skin or blood infections, endocarditis, meningitis, bone, and joint infections. The developed PLGA and PLA NPs could also offer a platform to (co)encapsulate other hydrophilic molecules such as amikacin, rifampin, or gentamicin. Moreover, synergic drug combinations could be loaded in the NPs. 

## Figures and Tables

**Table 1:** VCM encapsulation in NPs with different polymer compositions, effect on DL, EE, mean diameter, PDI, and Zeta potential. PLA and PLGA (co)polymers from EXPANSORB^®^ were used.

Name	M_w_ Range (kDa)	End Group	Lactic Unit (%)	Inherent Viscosity Range (dL/g)	DL (wt% + SD)	EE (wt% + SD)	Mean Diameter *^,†^ (nm)	PDI	Zeta Potential (mV + SD)
**PLA_8_C**	6–10	Carboxyl	100	0.05–0.20	7 ± 2	16 ± 5	319	0.18 ± 0.05	−1.6 ± 0.1
**PLA_15_C**	10–20	Carboxyl	100	0.15–0.30	<1	<1	322	0.25 ± 0.07	−1.7 ± 0.1
**PLGA_13_C**	5–20	Carboxyl	75	0.08–0.21	5 ± 1	11 ± 2	312	0.13 ± 0.01	−1.9 ± 0.1
**PLGA_15_C**	10–20	Carboxyl	50	0.15–0.25	14 ± 4	36 ± 2	325	0.19 ± 0.02	−1.9 ± 0.1
**PLGA_23_C**	15–30	Carboxyl	45	0.15–0.30	9 ± 1	24 ± 1	323	0.18 ± 0.03	−1.3 ± 0.1
**PLGA_28_C**	15–40	Carboxyl	50	0.25–0.40	8 ± 1	18 ± 2	326	0.21 ± 0.03	−0.7 ± 0.3
**PLGA_54_C**	42–65	Carboxyl	50	0.40–0.55	<1	<1	324	0.14 ± 0.01	−1.4 ± 0.3
**PLGA_61_C**	37–84	Carboxyl	75	0.38–0.64	<1	<1	350	0.16 ± 0.03	−1.4 ± 0.1
**PLGA_103_C**	76–130	Carboxyl	75	0.70–0.90	<1	<1	352	0.14 ± 0.01	−1.3 ± 0.3
**PLGA_113_C**	95–130	Carboxyl	50	0.65–0.90	<1	<1	367	0.17 ± 0.03	−1.4 ± 0.1
**PLGA_15_E**	10–20	Ester	50	0.15–0.25	<1	<1	323	0.26 ± 0.04	−0.9 ± 0.1
**PLGA_82_E**	72–91	Ester	50	0.60–0.70	<1	<1	344	0.15 ± 0.04	−1.4 ± 0.2

* Standard deviations (SD) are 25 nm for all the formulations. ^†^ Measured by DLS.

**Table 2:** Effect of the pH of VCM solution (inner aqueous phases, 1st emulsion) and of the suspension medium containing NaCl and the emulsifier PVA (outer aqueous phases, 2nd emulsion) on the incorporation of VCM and PLGA_15_C NP size and polydispersity.

pH of 1st Emulsion	pH of 2nd Emulsion	DL (wt% ± SD)	EE (wt% ± SD)	Mean Diameter *^,†^ (nm)	PDI
4.0	6.3	14 ± 4	36 ± 2	325	0.16 ± 0.17
	7.4	12 ± 1	36 ± 1	340	0.17 ± 0.04
	8.5	14 ± 4	43 ± 9	353	0.19 ± 0.06
7.4	6.3	22 ± 1	47 ± 2	318	0.15 ± 0.01
	7.4	25 ± 3	53 ± 7	312	0.15 ± 0.03
	8.5	23 ± 3	50 ± 7	319	0.19 ± 0.02

* Standard deviations (SD) are 25 nm for all the formulations. ^†^ Measured by DLS.

## Data Availability

Not applicable.

## Supplementary Materials

The following are available online at https://www.mdpi.com/article/10.3390/pharmaceutics13121992/s1. Experimental details: HPLC method, PVA quantification, effect of preparation parameters on DL, EE, and mean diameters, effect of the concentrations of NaCl and PVA on the incorporation of VCM and mean diameters. Figures: SEM image of VCM-loaded PLGA_15_C NPs, ^1^H and ^13^C MAS NMR spectra, STEM image of a VCM-loaded PLGA_15_C NP, SEC analysis, Cryo-TEM images of VCM-loaded PLGA_15_C NPs after 48 h incubation.
