# Supplementary material for: Compartmentalized Polymeric Nanoparticles Deliver Vancomycin in a pH-Responsive Manner
Source: Pharmaceutics. 2021 Nov 24;13(12):1992. doi: 10.3390/pharmaceutics13121992 (PMC8709497; doi:10.3390/pharmaceutics13121992)
Supplement: Supplementary file 1 [file pharmaceutics-13-01992-s001.zip › pharmaceutics-1450399-supplementary.pdf]

pharmaceutics

MDPI

Supplementary Materials: Compartmentalized Polymeric Nanoparticles Deliver Vancomycin in a pH-Responsive Manner

M. Seray Ural, Mario Menéndez-Miranda, Giuseppina Salzano, Jérémie Mathurin , Ece N. Aybeke, Ariane Deniset-Besseau, Alexandre Dazzi, Marianna Porcino, Charlotte Martineau-Corcos and Ruxandra Gref

HPLC method

The mobile A phase was water and the mobile B phase was acetonitrile, both containing 0.1% TFA. Chromatographic separation of VCM was performed at room temperature and elution was performed at a rate of 1 mL/min in gradient elution mode (Table S1). The injection volume was 10 µL.

Table S1. HPLC gradient elution program.

Time (min)	A (%)	B (%)
0	90	10
6	55	45
10	90	10

PVA quantification

1.3 g of NP suspension containing around 20 mg of $PLGA_{15}C$ or PLG_8C NPs was centrifuged at 17.000 x g for 15 min and the supernatant was discarded. The NP pellet was hydrolyzed overnight in 5 mL of 1N KOH and then neutralized with 1N HCl. To 0.1 mL of neutralized sample, 0.375 mL of 3.7% *w/v* boric acid solution, 0.01 mL of iodine solution (1.66% potassium iodide and 1.27% iodine in distilled water) *w/v* and 0.7 mL of 1N HCl were added. The absorbance of this solution was further measured in UV spectrometer at 620 nm. All samples were analyzed in triplicate.

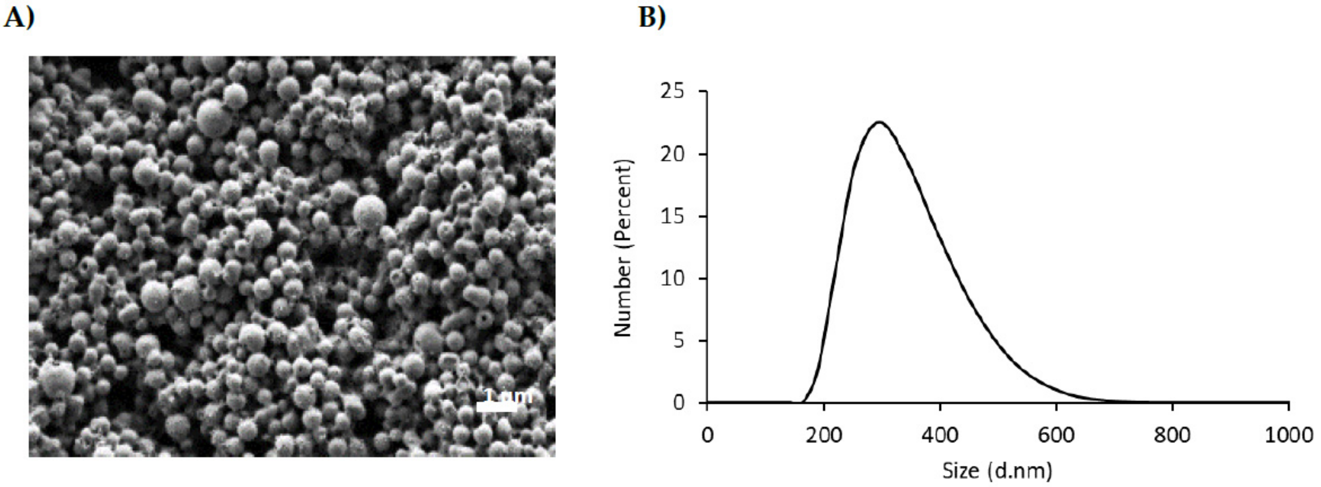

Figure S1. (**A**) Typical SEM image and (**B**) size distribution of $PLGA_{15}C$ NPs loaded with 14 wt% VCM.

Effect of preparation parameters on DL, EE and mean diameters

The influence of drug to polymer ratio on the DL and EE was investigated (Table S2). DL and EE increased with the drug/polymer ratio until reaching a plateau at the ratio of 0.4/1 (DL=14±4 wt%). In contrast, the drug/polymer ratio had no effect on the size of the NPs.

Table S2. Effect of drug/polymer mass ratio on DL, EE and mean diameter on VCM loaded PLGA$_{15}$C NPs.

Formulations (drug/polymer ratio)	DL (wt% ± SD)	EE (wt% ± SD)	Mean diameter*ł (nm)
0.2/1	9 ± 2	50 ± 11	324
0.3/1	10 ± 1	37 ± 4	330
0.4/1	14 ± 4	36 ± 2	320
0.5/1	14 ± 2	33 ± 3	327

* Standard deviations (SD) are 25 nm for all the formulations. ł Measured by DLS.

In complementary studies, it was found that the addition of NaCl during NP preparation had a high impact on VCM incorporation (Table S3). For example, DL increased from 9 ± 1 wt% to 14 ± 4 wt% in the presence of 1% NaCl and EE increased from 23 ± 1 % to 36 ± 2%, without having a significant effect on the NP size. Indeed, salts were reported to facilitate polymer precipitation and enhance DL. However, higher salt contents did not improve DL nor EE but led to much larger NPs (up to 460 nm).

Additionally, neither the use of more concentrated PVA solutions nor other emulsifiers lead to an improvement in DL. As presented in Table S3, doubling the concentration of PVA slightly affects the DL (from 12 ± 2 wt% to 14 ± 4 wt%). Noteworthy, the use of anionic surfactants such as sodium cholate instead of PVA failed to stabilize the emulsions, leading to phase separation.

Table S3. Effect of the concentrations of NaCl and PVA on the incorporation of VCM and mean diameters. NPs were prepared using PLGA$_{15}$C (co)polymer.

Formulations PVA/NaCl of outer phase	DL (wt% ± SD)	EE (wt% ± SD)	Mean diameter*ł (nm)
**0.25/0**	**9 ± 1**	**23 ± 2**	**318**
**0.25/1**	**12 ± 2**	**26 ± 2**	**320**
**0.25/5**	**11 ± 2**	**26 ± 2**	**496**
**0.5/0**	**9 ± 1**	**23 ± 1**	**320**
**0.5/1**	**14 ± 4**	**36 ± 2**	**320**
**0.5/5**	**14 ± 2**	**36 ± 2**	**460**

* Standard deviations (SD) are 25 nm for all the formulations. ł Measured by DLS.

NP Characterization by Solid-State NMR

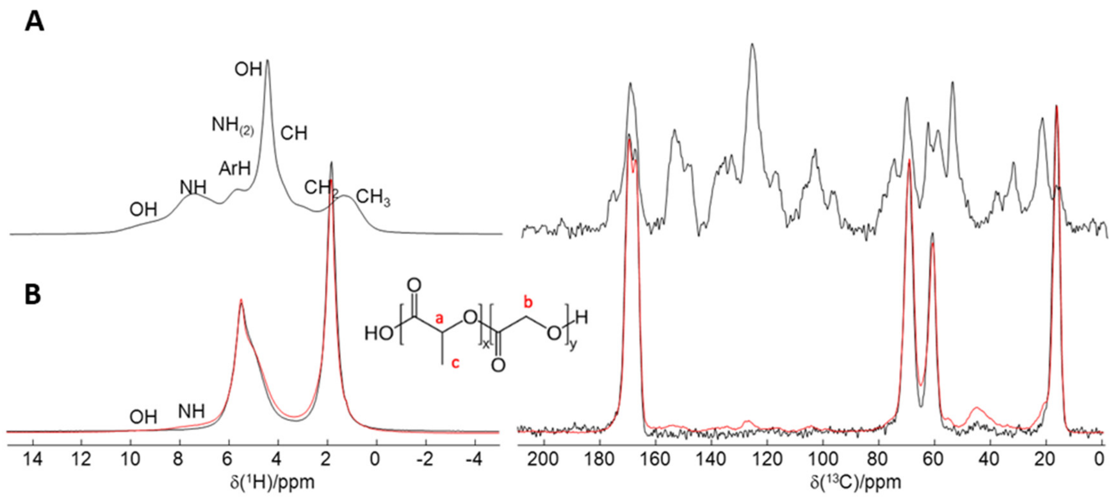

Figure S2. ^{1}H (left) and ^{13}C (right) MAS NMR spectra of (**A**) VCM and (**B**) empty PLGA15C NPs (black line) and VCM loaded PLGA15C NPs (red line).

STEM-EDX

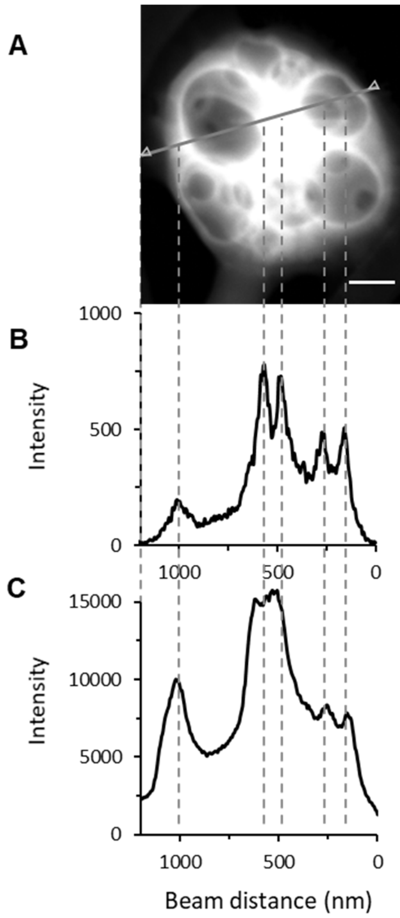

Figure S3. (**A**) STEM image of a VCM-loaded PLGA15C NP. EDX line scanning profiles of (**B**) Nitrogen and (**C**) Carbon. Nitrogen intensity at its peak values can be followed by the dashed lines. Scale bar is 250 nm.

Degradation studies

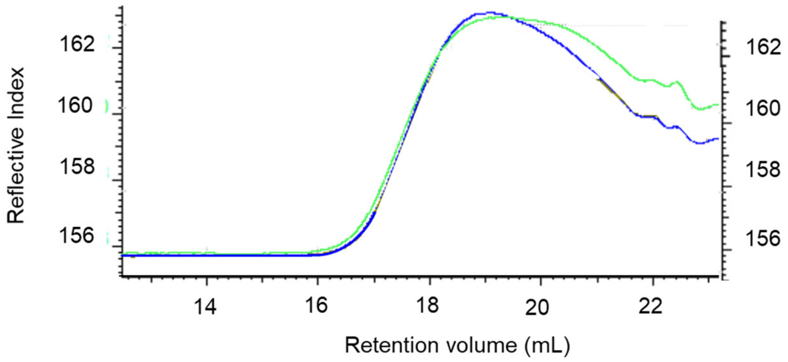

Figure S4. SEC analysis of loaded $PLGA_{15}C$ NPs incubated in phosphate buffer at pH 5.3 during 5 min (light green) and 4 days (blue). No significant polymer degradation was observed.

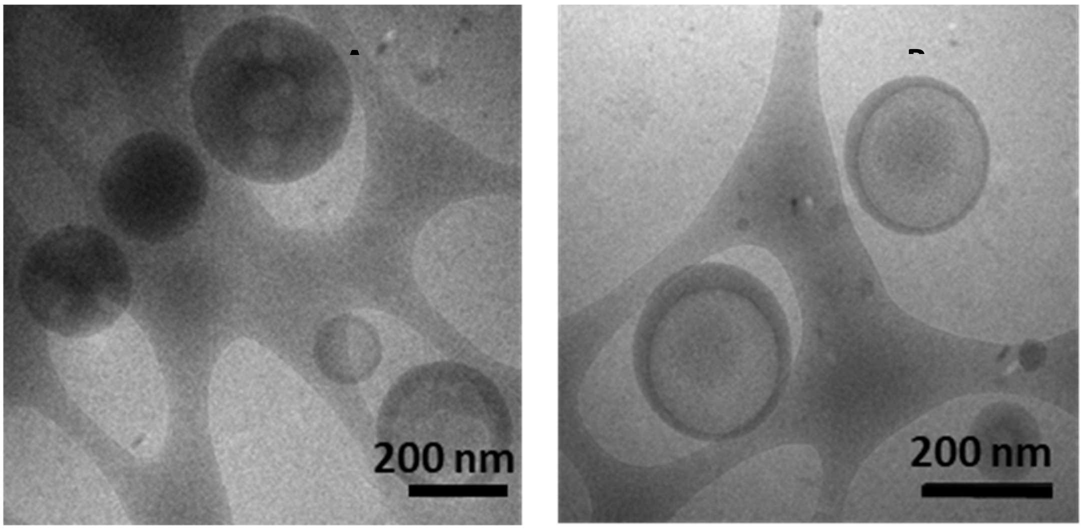

Figure S5. Typical Cryo-TEM images of VCM-loaded $PLGA_{15}C$ NPs after 48h incubation at 37°C: (**A**) in phosphate buffer at pH 5 and (**B**) in water.
